# libSBOLj3: a graph-based library for design and data exchange in synthetic biology

**DOI:** 10.1093/bioinformatics/btad525

**Published:** 2023-08-25

**Authors:** Göksel Mısırlı

**Affiliations:** School of Computer Science and Mathematics, Keele University, Keele, Staffordshire, ST5 5BG, United Kingdom

## Abstract

**Summary:**

The Synthetic Biology Open Language version 3 data standard provides a graph-based approach to exchange information about biological designs. The new data model has major updates and offers several features for software tools. Here, we present libSBOLj3 to facilitate data exchange and provide interoperability between computer-aided design and automation tools using this standard. The library adopts a graph-based approach. Tool developers can extend these graphs with application-specific information and use detailed validation reports to identify errors and interoperability issues and apply best practice rules.

**Availability and implementation:**

The libSBOLj3 library is implemented in Java and can be downloaded or used as a Maven dependency. The open-source project, code examples and documentation about accessing and using the library are available via GitHub at https://github.com/SynBioDex/libSBOLj3.

## 1 Introduction

The representation of data in standardized formats is ever more important for reproducibility and the development of predictable applications for synthetic biology. Data standards facilitate the use of different computer-aided design and automation tools, interoperability between these tools and the development of complex workflows (Mısırlı *et al.* 2017).

The Synthetic Biology Open Language (SBOL) is an open-source data standard to facilitate the electronic exchange of information about genetic parts, designs, and functional and experimental data. Several genetic design, visualization, modeling and automation tools and repositories use SBOL to represent and exchange data (Myers *et al.* 2017).

The recently developed SBOL3 (McLaughlin *et al.* 2020, Buecherl *et al.* 2023) adopts a fully graph-based approach in which SBOL entities represent nodes, and edges represent relationships between these entities. A linked data approach is used to identify and describe SBOL entities via existing ontologies and controlled vocabularies (Eilbeck *et al.* 2005, Lebo *et al.*, 2013, Mısırlı *et al.* 2019). SBOL3 has also been extended to support synthetic biology workflows more efficiently to build a web of design information.

A common approach to adopting biological data standards is the development of application programming interfaces for supporting different software tools, increasing interoperability between these tools and reducing the complexities of understanding and using data standards (Zhang *et al.* 2015). Here, we present libSBOLj3, a Java library for SBOL3. The library is document-centric and uses a graph-based approach to handle SBOL graphs efficiently. Tool developers can use this library to take advantage of the recent SBOL3 data standard, which provides increased reuse of biological design information and richer expressions to represent design-related constraints. Tool developers can also incorporate application-specific information and describe the provenance-based design, build, and test activities.

## 2 Results

The libSBOLj3 library fully implements the SBOL3 data model. Some of the important library features are summarized below.

### 2.1 Document-centric data exchange

**Figure 1.**
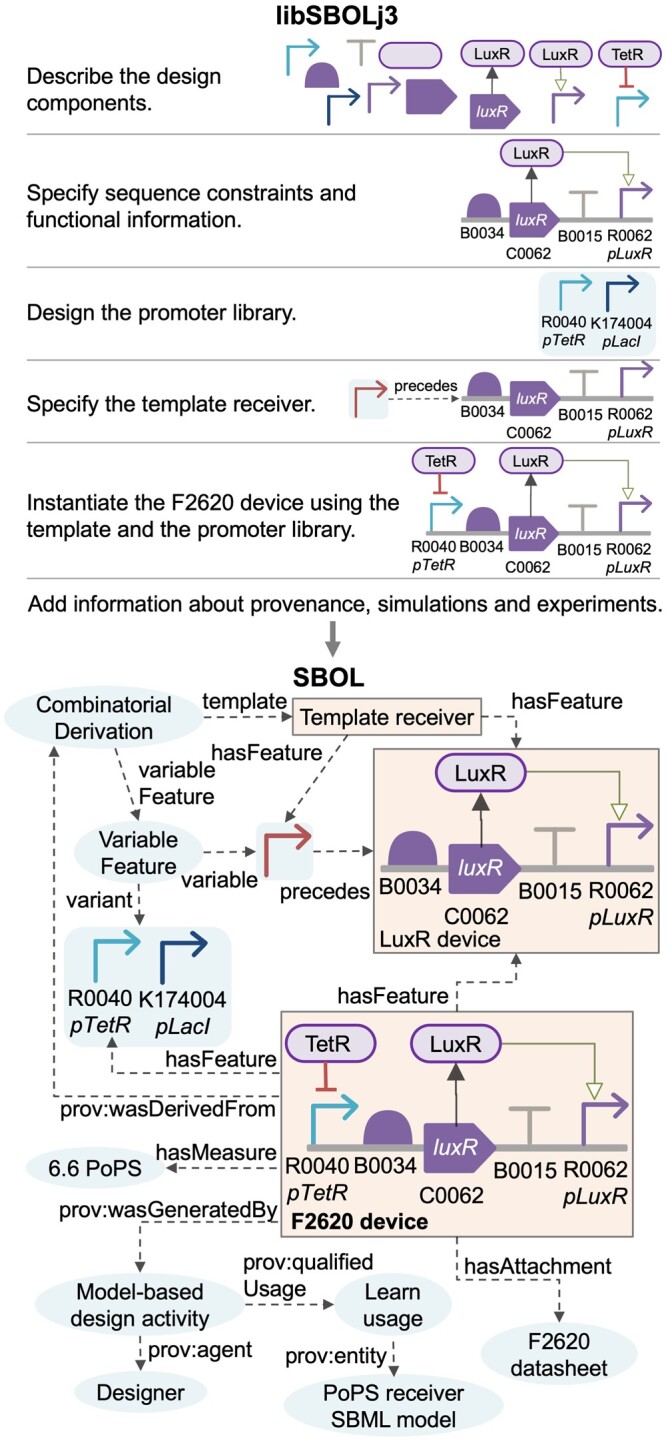
An example of using the libSBOLj3 library to design genetic circuits. The ‘Template receiver’, ‘LuxR device’, and ‘F2620 device’ designs are shown in boxes with solid lines. The F2620 design is derived from the template with features that can be varied combinatorially. Functional constraints about molecular interactions and structural constraints such as precedes and overlaps are specified. A datasheet about experimental results and provenance information about how this device is derived using a model-based design activity are associated with the design. A measurement entity with a polymerases per second (Canton *et al*. 2008) value is also included.

The library handles information as a single SBOL document, which can be constructed using programmatic entities designed to represent information of interest, such as a Component entity to represent a genetic part and a Sequence entity to define the composition of a part. These entities can further be described using child entities (Fig. 1). The library can write the resulting SBOL documents into files or in-memory variables and read them back.

### 2.2 Graph-based documents

Each SBOL document is associated with a graph, and the complexity of working with these graphs is hidden from the developers. These graphs can be serialized using four Resource Description Framework (RDF) formats (Allemang *et al.* 2020), recommended by the SBOL3 specification, and can also be processed by off-the-shelf graph tools to store, visualize, and query data. These formats are RDF/XML to support Extensible Markup Language (XML) tools, Turtle as a human-readable format, JavaScript Object Notation for Linked Data (JSON-LD) to use lightweight graphs for web applications, and N-Triples. The library includes methods to order N-triples graphs which can be helpful for versioning, comparing and streaming SBOL documents.

Each entity in libSBOLj3 is represented as a graph node and is associated with a unique identifier in the form of a uniform resource identifier (URI) or an internationalized resource identifier (IRI) consisting of a namespace and a local name. The library provides methods to control the creation of child entities with valid identifiers according to SBOL rules while offering flexibility to improve developers’ user experience.

### 2.3 Object-oriented application programming interface

The libSBOLj3 library adopts an object-oriented data manipulation and retrieval mechanism for a better user experience. Programmatic retrieval and update of SBOL entities and their properties are via simple getters and setters. Object properties and child objects are accessed when needed to support large and complex SBOL graphs. This lazy loading approach minimizes unnecessary interaction with the underlying graph. When an SBOL property is updated, the corresponding graph node is also updated.

The library provides additional graph-based access mechanisms for SBOL entities. An entity can be retrieved using its unique graph identifier via the getIdentified library call. The library also supports graph queries to search for entities. The search language is built on SPARQL SELECT. Prefix definitions and the SELECT clause are omitted, and a single search variable corresponding to SBOL entities with given criteria can be used. For example, the getIdentifieds method returns terminator genetic parts when it is executed with the “?identified a sbol:Component; sbol:role SO:0000141; sbol:type SBO:0000251.” query as input, and a list of Component objects is returned.

### 2.4 High-level application programming interface

The library has a high-level application programming interface (API) for repetitive or complex tasks and to decouple implementation details from the SBOL data model. Such methods can be used when design information relies on various entities and external ontologies or when describing complex workflows. These higher-level methods are regularly added based on community feedback.

### 2.5 Application-specific data support

The libSBOLj3 library supports integrating application-specific dataFor example, SBOL-specific objects can be annotated with key-value pairs using the library’s addAnnotation method. Moreover, the createMetadata method can be used to create application-specific objects.

### 2.6 Validation

Validation is an essential feature of the library to create valid SBOL documents. The SBOL standard lists several validation rules, some with multiple criteria involving different entities. The libSBOLj3 library validates SBOL documents for required and conformance-related rules and best practices. Validating best practices can be switched off programmatically. The library has options to can validate an SBOL document, an SBOL entity or a folder of SBOL documents. Invalid values and entities are reported with a list of validation messages which rely on SBOL-specific validation codes and descriptions. Contextual information about how an invalid value relates to a validated entity may involve a subgraph when multiple entities are involved. The library reports such a subgraph textually as a path between the validated entity and the invalid value in the form of a set of property names and values.

## 3 Methods

The libSBOLj3 library has been developed using the Java programming language and builds upon RDF graphs. The graph abstraction is achieved via the Jena (https://jena.apache.org) library, and the validation layer is built on Hibernate Validator (https://hibernate.org/validator). Project-specific dependencies and build automation are controlled with Maven (https://maven.apache.org). Test-driven development was adopted to create a user-friendly library and test validation rules effectively, providing more than 95% code coverage in unit tests.

## 4 Conclusion

The library presented here has been developed to facilitate interoperability between software tools in synthetic biology. Whilst the library can be used to work directly with the SBOL data model, the high-level API provides intuitive and less verbose methods to increase the user experience. Tool developers can use this library to create valid SBOL documents without the complexity of dealing with graphs and can take advantage of graph-based biological data integration, processing and querying.

## Data Availability

The data underlying this article are available on GitHub at https://github.com/SynBioDex/libSBOLj3.
